# Supplementary material for: Validation of the Ambivalence and Uncertainty Scale
Source: Int J Environ Res Public Health. 2025 Dec 29;23(1):46. doi: 10.3390/ijerph23010046 (PMC12841398; doi:10.3390/ijerph23010046)
Supplement: Supplementary file 1 [file ijerph-23-00046-s001.zip › Supplementary File _S5_Multi-group measurement invariance.pdf]

Supplementary File S5: Multi-group measurement invariance

Table E1. Multi-group measurement invariance (ordinal WLSMV, θ-parameterization)

Group	Model	χ^2	df	CFI	TLI	RMSEA	SRMR	ΔCFI	ΔRMSEA
**Gender**	Configural	358.254	54	0.984	0.978	0.100	0.062	—	—
	Thresholds	365.230	72	0.984	0.984	0.085	0.062	+0.001	−0.015
	Metric (thr+load)	376.830	80	0.984	0.986	0.081	0.062	0.000	−0.004
**Age**	Configural	443.543	135	0.981	0.975	0.101	0.074	—	—
	Thresholds	456.844	167	0.982	0.981	0.088	0.074	+0.001	−0.013
	Metric (thr+load)	516.310	199	0.981	0.982	0.084	0.076	−0.002	−0.004

Note. Δ values are relative to the immediately preceding (less constrained) model. Metric invariance for ordinal indicators corresponds to equal thresholds + loadings.
