# Supplementary material for: Validation of the Ambivalence and Uncertainty Scale
Source: Int J Environ Res Public Health. 2025 Dec 29;23(1):46. doi: 10.3390/ijerph23010046 (PMC12841398; doi:10.3390/ijerph23010046)
Supplement: Supplementary file 1 [file ijerph-23-00046-s001.zip › Supplementary File S1 AUS questionnaire original German version.pdf]

Supplementary File S1. Ambivalence and Uncertainty Scale (AUS) questionnaire original German version

Instruktion: Im Folgenden finden Sie einige Aussagen zu möglichen herausfordernden Gefühlen und Situationen. Bitte kreuzen Sie zu jeder Aussage an, inwiefern diese in den letzten zwei Wochen, einschließlich heute, auf Sie persönlich zutreffen.

		Trifft gar nicht zu	Trifft eher nicht zu	Trifft eher zu	Trifft vollständig zu
	**Question**	1	2	3	4
1	Ich erlebe meine Gefühle als widersprüchlich.				
2	Widersprüchliche Gefühle/Gedanken zu tolerieren fällt mir schwer.				
3	Ich bin froh, wenn Entscheidungen für mich getroffen werden.				
4	Ich zweifle oft, ob sich die Anstrengung für eine Sache lohnt.				
5	Zwischen attraktiven Optionen zu wählen, fällt mir schwer.				
6	Unsicherheit zu ertragen, fällt mir schwer.				
7	Ich weiß oft nicht, was ich will.				
8	Zwischen zwei unangenehmen Alternativen zu wählen, fällt mir schwer.				
9	Ich fühle mich oft zwischen Optionen/Perspektiven hin- und hergerissen				
