# Supplementary material for: Validation of the Ambivalence and Uncertainty Scale
Source: Int J Environ Res Public Health. 2025 Dec 29;23(1):46. doi: 10.3390/ijerph23010046 (PMC12841398; doi:10.3390/ijerph23010046)
Supplement: Supplementary file 1 [file ijerph-23-00046-s001.zip › Supplementary File _S2_Additional_factor-retention_diagnostics.pdf]

Supplementary File S2: Additional factor-retention diagnostics

Setup. Factor-retention tests were conducted on the **polychoric correlation matrix** from the EFA. To ensure numerical stability, we also evaluated a **smoothed** version of the matrix.

B1. Parallel Analysis (PA)

- **Method.** PA on the (smoothed) polychoric matrix using principal axis factoring.
- **Result.** PA suggested **up to four factors** (a known over-extraction tendency with ordinal/polychoric data).

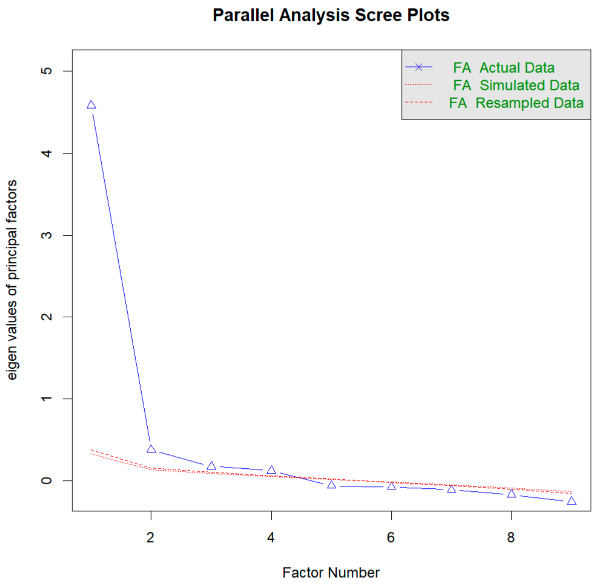

Figure B1. *Parallel Analysis plot on the smoothed polychoric matrix.*

B2. Velicer's Minimum Average Partial (MAP)

- **Method.** MAP on the smoothed polychoric matrix.
- **Result. Minimum at 1 factor**; MAP sequence (rounded): **.038**, .052, .082, .113, .202, .329, .463, 1.000, NA.
  → Interpreted as support for a **unidimensional** solution.

B3. Very Simple Structure (VSS; c1/c2)

- **Method.** VSS (complexity 1 and 2) on the smoothed polychoric matrix.
- **Result.** Numeric output was unstable due to near-singularity; however, the **VSS diagnostics** showed only **trivial incremental gains beyond 1–2 factors** with an early elbow in SRMR – again consistent with a **dominant first factor**.

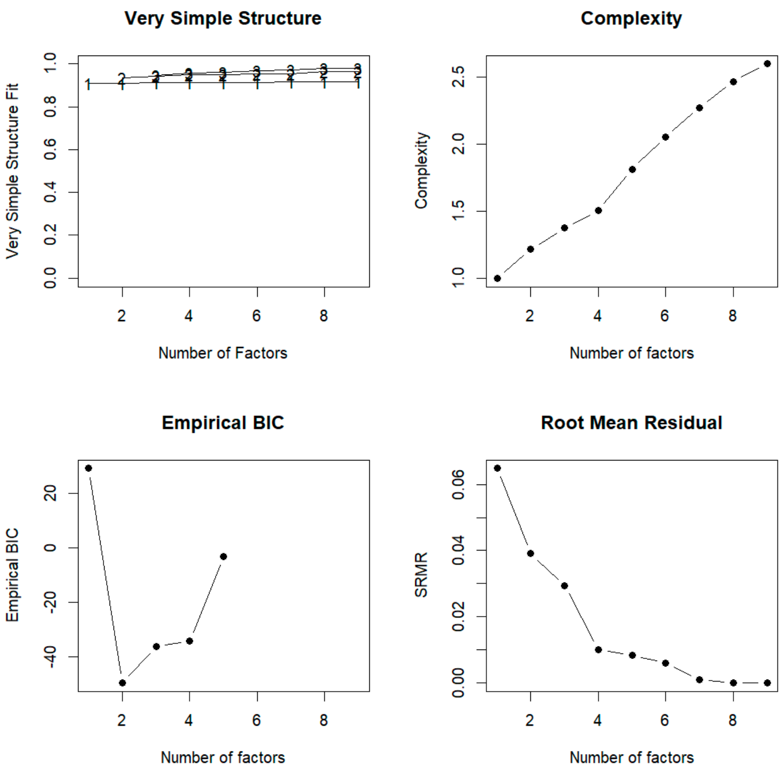

Figure B2. *VSS diagnostics plot on the smoothed polychoric matrix.*

B4. Decision

Across tests (**PA**, **MAP**, **VSS**), the evidence converged on a **one-factor** structure (with PA prone to over-extraction in this context). Consequently, **no rotation** was applied in the EFA.
