# Supplementary material for: Validation of the Ambivalence and Uncertainty Scale
Source: Int J Environ Res Public Health. 2025 Dec 29;23(1):46. doi: 10.3390/ijerph23010046 (PMC12841398; doi:10.3390/ijerph23010046)
Supplement: Supplementary file 1 [file ijerph-23-00046-s001.zip › Supplementary File _S3_Split-sample cross-validation.pdf]

Supplementary File S3: Split-sample cross-validation (EFA → CFA)

Design. The full sample (N = 1,133) was randomly split into an **EFA set** (n = 676) and a **CFA holdout** (n = 457). Items were 4-point ordinal.

EFA (polychoric PAF; one factor; no rotation). Sampling adequacy was excellent (KMO = .90). Bartlett's test indicated factorability, $\chi^2(36) = 3235.93$, $p < .001$. A one-factor solution explained **51.2%** of the variance (bootstrap median; 95% CI [.473, .549]). Standardized loadings (point estimate; 95% bootstrap CI; B = 500) were:

Item	Item	Item
Item 9	0.857	[0.816, 0.889]
Item 7	0.797	[0.751, 0.844]
Item 5	0.702	[0.647, 0.755]
Item 8	0.694	[0.630, 0.744]
Item 1	0.689	[0.623, 0.747]
Item 2	0.683	[0.616, 0.743]
Item 6	0.680	[0.619, 0.743]
Item 4	0.647	[0.472, 0.632]
Item 3	0.646	[0.446, 0.627]

CFA (holdout; WLSMV, θ-parameterization). Global fit indices were **CFI = .979**, **TLI = .971**, **SRMR = .065**, **RMSEA = .105**, $\chi^2(27) = 161.98$, $p < .001$. Bootstrapped (case-resampling, B = 500) 95% CIs for standardized loadings:

Item	Item	Item
Item 9	0.812	[0.760, 0.865]
Item 7	0.766	[0.700, 0.829]
Item 2	0.748	[0.670, 0.821]
Item 1	0.709	[0.634, 0.782]
Item 1	0.634	[0.551, 0.715]
Item 8	0.626	[0.546, 0.704]
Item 6	0.623	[0.535, 0.706]
Item 4	0.557	[0.472, 0.632]
Item 3	0.541	[0.446, 0.627]

Bootstrapped fit-index quantiles (2.5% / 50% / 97.5%): **CFI** .953 / .975 / .988; **TLI** .938 / .967 / .984; **RMSEA** .083 / .114 / .149; **SRMR** .054 / .071 / .090.

Conclusion. Convergent EFA and holdout-CFA results, together with narrow bootstrapped CIs for item loadings, **support a unidimensional measurement model**. CFI/TLI and SRMR indicate good to acceptable fit; RMSEA was higher, which is common in single-factor, low-df models with ordinal indicators. Overall, the evidence favors a **robust one-factor structure**.
