# Supplementary material for: Validation of the Ambivalence and Uncertainty Scale
Source: Int J Environ Res Public Health. 2025 Dec 29;23(1):46. doi: 10.3390/ijerph23010046 (PMC12841398; doi:10.3390/ijerph23010046)
Supplement: Supplementary file 1 [file ijerph-23-00046-s001.zip › Supplementary File _S4_EFA_variants.pdf]

Supplementary File S4: EFA variants (2- and 3-factor models)

Method. To evaluate whether solutions with more than one factor would improve interpretability, we estimated **polychoric PAF** models with (a) **oblimin** rotation (primary) and (b) **varimax** (sensitivity), using the same 9 AUS indicators as in the main EFA. Interpretability rules were pre-specified: primary loading ≥ .40, cross-loading difference ≥ .20, and ≥ 3 salient items per factor.

Summary. The **2-factor oblimin** model showed **high factor intercorrelation** (Φ = .663) and **3 items** with cross-loadings ≥ .30; the **3-factor oblimin** model likewise yielded **substantial factor correlations** (Φs = .601/.420/.292) and a cross-loading on one item. Sensitivity **varimax** solutions produced **more** cross-loadings (2F: 5; 3F: 7). Together with MAP/VSS/PA (Appendix B) and the holdout CFA (Appendix C), these findings indicate a **dominant general factor**; multi-factor solutions offer no substantive advantage over the unidimensional model.

Factor intercorrelations (oblimin).
2-factor: Φ_{12} = **.663**.
3-factor: Φ_{12} = **.601**, Φ_{13} = **.420**, Φ_{23} = **.292**.

Table D1. Rotated loadings (polychoric PAF, 2 factors, oblimin)

Item	F1	F2	h²	Cross-loads ≥.30
8	0.897	−0.157	0.642	1
5	0.732	0.015	0.551	1
7	0.703	0.150	0.655	1
9	0.602	0.322	0.723	2
3	0.518	0.174	0.418	1
6	0.384	0.359	0.459	2
1	−0.003	0.828	0.682	1
2	0.048	0.757	0.623	1
4	0.347	0.361	0.416	2

Table D2. Factor intercorrelation matrix Φ (2-factor, oblimin)

	F1	F2
F1	1.000	0.663
F2	0.663	1.000

Table D3. Rotated loadings (polychoric PAF, 3 factors, oblimin)

Item	F1	F2	F3	h²	Cross-loads ≥.30
8	0.853	−0.170	0.058	0.621	1
7	0.788	0.121	−0.082	0.696	1
9	0.717	0.293	−0.101	0.784	1
5	0.691	−0.060	0.174	0.556	1

Item	F1	F2	F3	h²	Cross-loads ≥.30
3	0.499	0.082	0.182	0.424	1
4	0.379	0.269	0.117	0.409	1
1	0.062	0.869	−0.027	0.809	1
2	0.033	0.596	0.365	0.651	2
6	0.290	0.187	0.490	0.597	1

Table D4. Factor intercorrelation matrix Φ (3-factor, oblimin)

	F1	F2	F3
F1	1.000	0.601	0.420
F2	0.601	1.000	0.292
F3	0.420	0.292	1.000

Sensitivity (orthogonal rotation)

Table D5. Rotated loadings (polychoric PAF, 2 factors, varimax)

Item	F1	F2	h²	Cross-loads ≥.30
8	0.777	0.196	0.642	1
7	0.697	0.411	0.655	2
5	0.681	0.295	0.551	1
9	0.660	0.535	0.723	2
3	0.535	0.363	0.418	2
1	0.266	0.781	0.682	1
2	0.290	0.734	0.623	1
6	0.471	0.487	0.459	2
4	0.438	0.474	0.416	2

Table D6. Rotated loadings (polychoric PAF, 3 factors, varimax)

Item	F1	F2	F3	h²	Cross-loads ≥.30
8	0.735	0.128	0.254	0.621	1
7	0.721	0.380	0.179	0.696	2
9	0.694	0.517	0.187	0.784	2
5	0.633	0.173	0.353	0.556	2
3	0.496	0.241	0.346	0.424	2
4	0.421	0.377	0.299	0.409	2
1	0.252	0.831	0.233	0.809	1
2	0.226	0.559	0.536	0.651	2
6	0.382	0.262	0.619	0.597	2

Cross-loading counts (≥ .30): 2F-oblimin = **3**, 3F-oblimin = **1**, 2F-varimax = **5**, 3F-varimax = **7**.

Interpretation. Both the **magnitude of Φ** (up to .66 in 2F; .60/.42/.29 in 3F) and the **number of cross-loadings** indicate that multi-factor solutions largely reflect a strong **general factor**, consistent with the **1-factor** model retained in the main text.

Decision for one-factor solution (key reasons):

- **Factor retention converges on 1 factor:** Velicer's **MAP minimum at 1** (MAP_1 = **.038**); PA can over-factor with polychorics and few items – see Appendix B for details.
- **Strong general saturation in multi-factor trials: High factor intercorrelations** (**2F Φ_{12} = .663; 3F Φs = .601/.420/.292**) indicate overlapping dimensions rather than distinct subscales.
- **Cross-loadings undermine simple structure: 2F-oblimin = 3**, **3F-oblimin = 1**, and more under varimax (**2F = 5**, **3F = 7**), arguing against clean 2–3 factor solutions.
- **Substantial general loadings & variance explained:** EFA loadings **.65–.86**; bootstrap **Proportion Var = .512** (95% CI **[.473, .549]**), supporting a robust common factor.
- **Holdout CFA supports 1 factor: CFI = .979**, **TLI = .971**, **SRMR = .065** (good/acceptable); **RMSEA = .105** is expectedly inflated for single-factor, low-df ordinal models.
- **Parsimony & interpretability:** A single composite score makes statistically sense and is practical; multi-factor variants add complexity with **limited incremental psychometric gain** in this dataset.
